# Anti-Hexokinase 1 Antibody as a Novel Serum Biomarker of a Subgroup of Diabetic Macular Edema

**DOI:** 10.1038/s41598-019-39777-z

**Published:** 2019-03-18

**Authors:** Tatsuya Yoshitake, Tomoaki Murakami, Shin Yoshitake, Kiyoshi Suzuma, Yoko Dodo, Masahiro Fujimoto, Shinji Ito, Akitaka Tsujikawa

**Affiliations:** 1 Department of Ophthalmology and Visual Sciences, Kyoto University Graduate School of Medicine, Kyoto, Japan; 2 Medical Research Support Center, Graduate School of Medicine, Kyoto University, Kyoto, Japan

## Abstract

Diabetic retinopathy (DR) induces the breakdown of the blood-retinal barrier and promotes neuroinflammation, although autoimmune responses to sequestered retinal antigens remain poorly understood. In this study, we investigated the autoantibodies for retinal antigens in sera from diabetic macular edema (DME) patients. Screening by immunoblotting demonstrated that IgG from 7 of 10 DME sera samples reacted to an ~102-kDa autoantigen from porcine retinas. Immunoprecipitation with autoantibodies from DME sera and subsequent mass spectrometry enabled us to identify hexokinase 1 as an autoantigen reactive to IgG from DME sera. IgG in 7 of 10 DME sera partially colocalized to hexokinase 1 in the outer plexiform layer of rodent retinas. Quantitative analyses using enzyme-linked immunosorbent assays revealed that the serum titers of this autoantibody were significantly higher in the DME sera than those in the sera from diabetic patients without DME, and 20 (24.1%) of the 83 DME serum samples had higher IgG titers than the cutoff value (mean + 2 standard deviations of the sera from diabetic patients without DR). Multivariate logistic regression analysis confirmed that the higher titer of anti-hexokinase 1 IgG was clinically feasible for the diagnosis of DME. These data identify anti-hexokinase 1 antibody as a serum biomarker of a subset of DME.

## Introduction

Diabetic retinopathy (DR), a subset of diabetic microangiopathy, often leads to severe visual impairment worldwide^1–3^. Diabetes promotes the breakdown of the blood-retinal barrier (BRB) and subsequently induces edematous changes and dysfunction in neuroglial components in diabetic macular edema (DME)^4^. Recent advances in molecular biology and clinical trials have resulted in the clinical application of anti-vascular endothelial growth factor (VEGF) treatment for DME, which has had a significant impact on visual outcomes^5–9^. This result implies that better screening for or diagnosis of DME would improve the visual prognosis in diabetic patients.

Patients with diabetes mellitus (DM) routinely undergo blood sampling in the internal medicine clinic. Several blood biomarkers, e.g., fasting blood glucose and hemoglobin A1c (HbA1c), are used to monitor the clinical efficacy of systemic treatments or indicate the necessity of further examination for diabetic complications^10,11^. In particular, several serum biomarkers of DR are related to biochemical pathways, glucose metabolism, inflammation, microRNA, and the proteome^12–17^. Diabetic patients who visit the internal medicine clinic are not necessarily referred to the eye clinic and are not necessarily examined for vision-threatening DR^1^. These concerns suggest the necessity of identifying blood biomarkers of proliferative diabetic retinopathy (PDR) or DME that should be adequately treated by ophthalmologists.

Recent investigations have elucidated immunological aspects of chorioretinal vascular diseases^18,19^. The inner and outer BRBs, which are composed of retinal vascular endothelial cells and retinal pigment epithelium (RPE), respectively, sequester retinal autoantigens to prevent autoimmune disease development^20–23^. In chorioretinal vascular diseases, e.g., age-related macular degeneration (AMD) and DR, vascular hyperpermeability or the disruption of properties of the RPE barrier allows immunological agents to react to the sequestered antigens. Autoantibodies against retinal antigens are subsequently induced, and innate immunity is activated in AMD^19,24–26^. In addition to their contribution to the pathogenesis of chorioretinal vascular diseases, these antibodies indicate retinal impairment and can be biomarkers of these diseases^24,25,27^. Diabetes also modulates and is modulated by autoimmunity^28^, and several autoantibodies have been identified as serum biomarkers of diabetic complications^29,30^. However, antigens against autoantibodies from DME or PDR sera remain ill-defined.

In the current study, we identified anti-hexokinase 1 antibody as a novel serum autoantibody using a combination of biochemistry and mass spectrometry, and we investigated the association between the antibody titer and clinical parameters in DME.

## Results

### Screening of autoantibodies in DME sera

**Figure 1:**
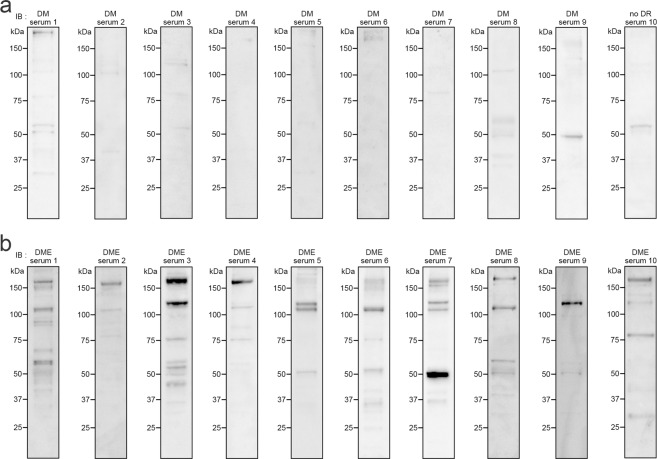
Immunoreactivity of DME sera to retinal lysates. Immunoblotting was performed to determine the immunoreactivity of serum IgG in 10 serum samples from the DM group (**a**) or DME group (**b**) to porcine retinal antigens. The patients’ characteristics are shown in Table 1.

We first screened for anti-retinal autoantibodies in DME sera using immunoblotting for porcine total retinal lysates and found that 7 (70.0%) of the 10 DME sera samples showed immunoreactivity to an ~102-kDa antigen, whereas there were only minimal levels of band intensities reactive to DM sera (Fig. 1, Table 1). Additional immunofluorescence analysis using patients’ sera as primary antibodies revealed that 7 (70.0%) of the 10 DME sera exhibited immunoreactivity to autoantigens in the outer plexiform layer (OPL) of C57BL/6 J mice (data not shown).

**Table 1:** Characteristics of patients whose sera were analyzed by Western blotting.

Characteristics	DM group (n = 10)	DME group (n = 10)
Age (years)	60.8 ± 12.0	69.2 ± 5.9
Gender (male/female)	6/4	6/4
Diabetes duration (years)	8.8 ± 7.3	14.3 ± 8.0
Mean arterial blood pressure (mmHg)	92.2 ± 3.8	98.2 ± 13.1
HbA1c (%)	8.09 ± 1.44	7.39 ± 1.62
Systemic hypertension	7	8
Dyslipidemia	6	5
Phakic in both eyes	9	8
International classification		
No apparent retinopathy	10	—
Mild NPDR	—	0
Moderate NPDR	—	5
Severe NPDR	—	3
PDR	—	2
Prior PRP in either eye	—	4

### Identification of hexokinase 1 as a target of DME autoantibodies

**Figure 2:**
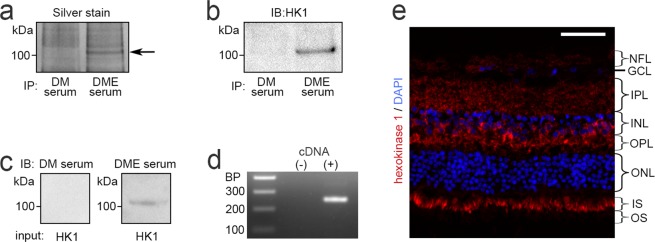
Identification of anti-hexokinase 1 antibody as an autoantibody in DME serum. (**a**) Retinal autoantigens immunoprecipitated with serum IgG of the DM group or DME group were analyzed by SDS-PAGE and silver staining. Subsequent mass spectrometry revealed that the ~102-kDa unique band (arrow in panel a) corresponded to hexokinase 1, which was confirmed by immunoblotting with rabbit monoclonal anti-hexokinase 1 antibody (**b**). The patients’ characteristics are shown in Supplementary Table S1. (**c**) Human recombinant hexokinase 1 was immunoblotted with IgG from DM or DME serum. (**d**) PCR analysis to determine the expression of hexokinase 1 mRNA in human retinas. (**e**) High levels of hexokinase 1 expression in the OPL, the outer half of the INL, and IS and, to a lesser extent, in the NFL and IPL in human retinas. Scale bar, 50 μm. GCL, ganglion cell layer; HK1, hexokinase 1; INL, inner nuclear layer; IPL, inner plexiform layer; IS, inner segment; NFL, nerve fiber layer; ONL, outer nuclear layer; OPL, outer plexiform layer; OS, outer segment.

To identify the antigens reactive to the autoantibodies, we performed immunoprecipitation using DME sera and analyzed the precipitated proteins by subsequent mass spectrometry (Fig. 2a, Supplementary Table S1). The mass spectrometry revealed that the ~102-kDa precipitated antigen was hexokinase 1. We then confirmed that the precipitated protein was reactive to a commercially available anti-hexokinase 1 antibody and that the same sera from DME patients showed immunoreactivity to recombinant hexokinase 1 protein (Fig. 2b,c). We thus identified anti-hexokinase 1 antibody as one of the autoantibodies from DME sera.

**Figure 3:**
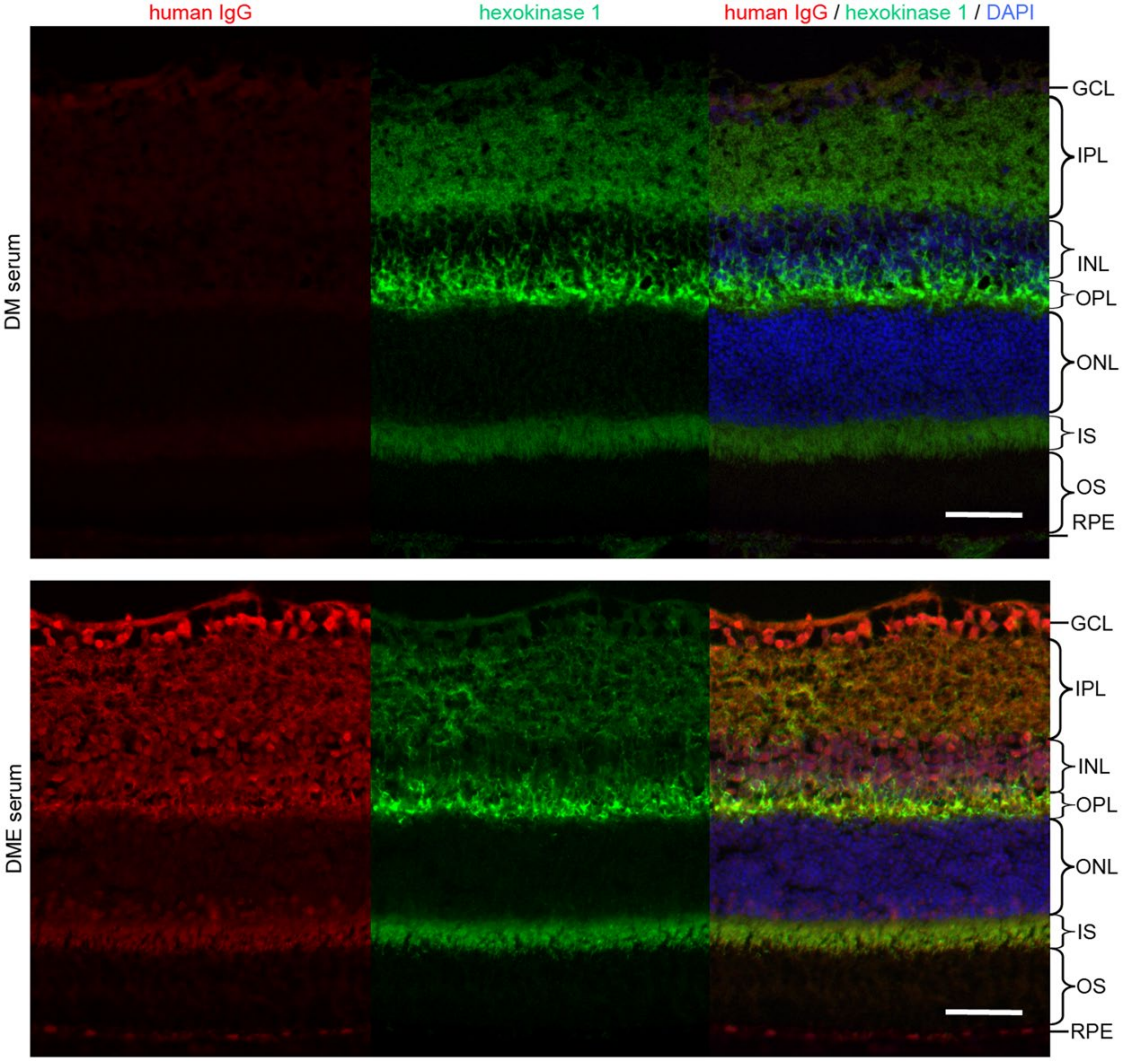
Localization of autoantigens from serum IgG with a high titer of anti-hexokinase 1 antibody. Immunofluorescence of serum IgG from representative cases of the DM and DME groups, which corresponded to the DM serum 4 and DME serum 10 in the Western blot experiements (Fig. 1), respectively (Supplementary Table S2). Fluorescence signals of serum IgG from DME patients were partially colocalized to hexokinase 1 in the OPL, IPL and IS of C57BL/6 J mice. Scale bar, 50 μm.

Hexokinase 1, one of four major isozymes, mediates glucose phosphorylation, the first step in the glucose metabolism pathway, and is ubiquitously expressed and localizes to the surface of the mitochondrial outer membrane^31^. Polymerase chain reaction (PCR) analysis showed that hexokinase 1 mRNA was expressed in the human retinas (Fig. 2d). Immunofluorescence staining revealed that hexokinase 1 was highly localized to the OPL, the outer half of the inner nuclear layer (INL), and the photoreceptor inner segment (IS) and, to a lesser extent, to the nerve fiber layer (NFL) and the inner plexiform layer (IPL) in human retinas (Fig. 2e). The mRNA levels and the protein localization were similar in the retinas of C57BL/6 J and Ins2Akita mice (Supplementary Fig. S1)^32^. The immunoreactivity of IgG from DME serum with high titers of anti-hexokinase 1 antibody was often colocalized with hexokinase 1 in the OPL and to some extent in the IPL of the mouse retinas (Fig. 3, Supplemenatary Table S2).

### Quantitative analyses of serum titers of anti-hexokinase 1 antibody

**Figure 4:**
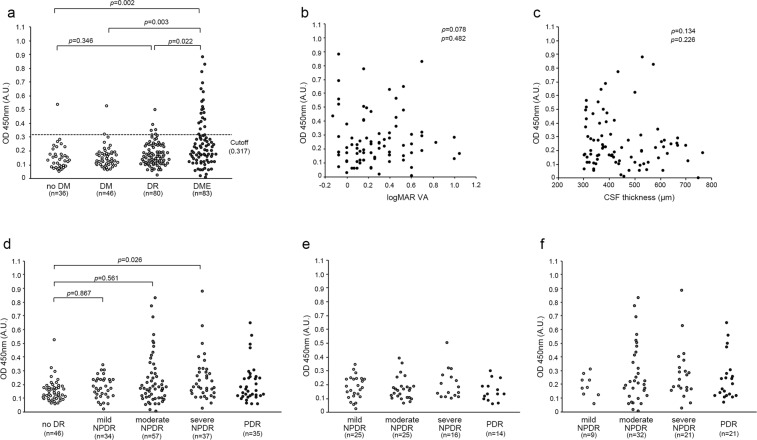
Quantification of the titer of anti-hexokinase 1 antibody in DME sera. (**a**) ELISA revealed that the titers of anti-hexokinase 1 IgG were higher in the DME group than in no DM group, DM group, or DR group. (**b**,**c**) In 83 cases in the DME group, the titer of anti-hexokinase 1 antibody was not associated with either logMAR VA or CSF thickness. (**d**–**f**) The titer was associated with individual grades of DR severity in all diabetic cases (**d**), the DR group (**e**), and the DME group (**f**). The titers were not different between individual DR severity grades, although there was a trend toward higher titers in patients with moderate or severe NPDR in the DME group.

Quantitative investigation using enzyme-linked immunosorbent assay (ELISA) revealed that the titer of anti-hexokinase 1 antibody in sera from patients with center-involved DME defined by optical coherence tomography (OCT) measurement (the DME group) was significantly higher than in nondiabetic subjects (the no DM group), patients with type 2 DM but not DR (the DM group), or patients with DR but not center-involved DME (the DR group) (*P* = 0.002, *P* = 0.003, or *P* = 0.022, respectively; Fig. 4a, Table 2, Supplementary Fig. S2, Table S3). Twenty (24.1%) of the 83 DME sera samples had higher titers of this autoantibody than the threshold of 2 standard deviation (SD) above the mean value in the DM group. We investigated 83 eyes with DME and found that the titers of this autoantibody were not related to the logarithm of the minimum angle of resolution visual acuity (logMAR VA) or central subfield (CSF) thickness (Fig. 4b,c). Additional analyses showed a trend toward higher titers in participants with moderate nonproliferative diabetic retinopathy (NPDR) or more severe grades, and the autoantibody titer in patients with severe NPDR was higher than in diabetic patients without DR (Fig. 4d, Supplemenatary Table S4). However, there were no significant differences between individual severity grades in the eyes in the DR group (Fig. 4e). The titer was correlated to the age of participants (ρ = 0.199, *P* = 0.004), whereas there were no association with other systemic or ocular factors in all 209 diabetic cases.

**Table 2:** Characteristics of the patients whose sera were quantified using ELISA.

Characteristics	no DM group (n = 36)	DM group (n = 46)	DR group (n = 80)	DME group (n = 83)	*P*-value
Age (years)	75.5 ± 7.70	63.5 ± 11.0*	62.7 ± 11.9*	64.1 ± 12.2*	<0.001
Gender (male/female)	11/25	32/14*	58/22*	54/29*	<0.001
Diabetes duration (years)	—	11.3 ± 9.8	16.1 ± 9.6^†^	12.3 ± 8.2	0.009
Mean arterial blood pressure (mmHg)	87.8 ± 8.1	90.5 ± 7.4	93.8 ± 14.3	96.8 ± 11.7*^†^	0.001
HbA1c (%)	—	7.76 ± 1.44	7.94 ± 1.59	7.85 ± 1.65	0.814
Systemic hypertension	21	25	52	55	0.515
Dyslipidemia	15	25	41	43	0.689
Phakic in both eyes	35	41	55*^‡^	56*^‡^	<0.001
International classification					
No apparent retinopathy	—	46	—	—	
Mild NPDR	—	—	25	9	
Moderate NPDR	—	—	25	32	
Severe NPDR	—	—	16	21	
PDR	—	—	14	21	
Prior PRP in either eye	—	—	12	31	0.001

*P < 0.01 vs. no DM group; ^†^*P* < 0.05 vs. DM group; ^‡^*P* < 0.01 vs. DM group.

**Figure 5:**
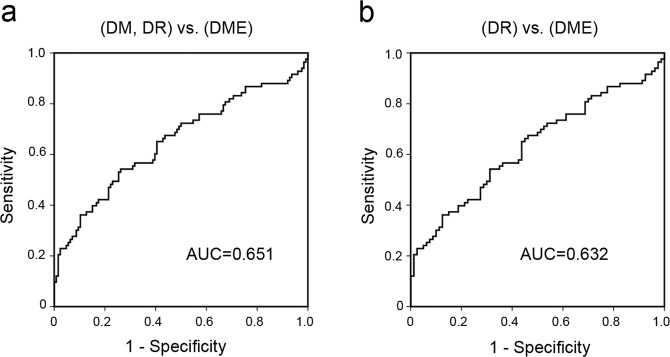
ROC curves for individual DR status of the titer of anti-hexokinase 1 antibody. The ROC curve for DME diagnosis of the titer of anti-hexokinase 1 IgG discriminating the DME group from no DME group in all 209 cases encompassing the DM, DR, and DME groups (**a**) or the 163 cases encompassing the DR and DME groups (**b**).

We recently identified anti-fumarase IgG in the sera from a subgroup of DME patients and therefore investigated the relationship between these autoantibodies^33^. There was a moderate correlation between the serum titers of anti-hexokinase 1 IgG and anti-fumarase IgG in 83 cases of the DME group (*ρ* = 0.341, *P* = 0.002). Multivariate logistic regression analysis using systemic factors demonstrated that the serum titers of anti-hexokinase 1 IgG and anti-fumarase IgG were the significant predictors of center-involved DME in all 209 cases of type 2 DM or 163 cases with DR (Tables 3, 4). By contrast, the areas under the receiver operating characteristic (ROC) curve (AROC) for DME cases of the serum titer of anti-hexokinase 1 IgG was 0.651 (95% confidence interval [CI], 0.546–0.710) or 0.632 (95% CI, 0.546–0.717) in patients with diabetes or DR, respectively (Fig. 5a,b).

**Table 3:** Multivariate logistic regression analysis to determine the factors associated with center-involved DME in 209 diabetic patients.

Characteristics	Univariate		Multivariate	
	OR (CI 95%)	*P* value	OR (CI 95%)	*P* value
Age	1.008 (0.985–1.032)	0.502	—	—
Gender	1.343 (0.741–2.432)	0.331	—	—
Diabetes duration	0.977 (0.946–1.010)	0.164	—	—
Mean arterial blood pressure	1.029 (1.004–1.055)	0.024	1.031 (1.001–1.061)	0.039
HbA1c	0.987 (0.821–1.186)	0.885	—	—
Systemic hypertension	1.250 (0.701–2.230)	0.450	—	—
Dyslipidaemia	0.961 (0.551–1.675)	0.888	—	—
Phakic in both eyes	1.543 (0.834–2.855)	0.167	—	—
PDR in either eye	2.710 (1.288–5.702)	0.009	0.787 (0.273–2.266)	0.657
Prior PRP in either eye	5.663 (2.695–11.902)	<0.001	8.011 (2.828–22.697)	<0.001
Titer of anti-fumarase IgG	2077.4 (48.6–88862.3)	<0.001	266.7 (3.0–23470.4)	0.014
Titer of anti-hexokinase 1 IgG	210.3 (16.5–2679.3)	<0.001	183.8 (8.7–3889.9)	<0.001

Univariate logistic regression or Multivariate logistic analysis using significant parameters (Wald’s chi-square > 2) as independent parameters.

**Table 4:** Multivariate logistic regression analysis to determine the factors associated with center-involved DME in 163 DR participants.

Characteristics	Univariate		Multivariate	
	OR (CI 95%)	*P* value	OR (CI 95%)	*P* value
Age	1.010 (0.984–1.036)	0.452	—	—
Gender	1.416 (0.727–2.758)	0.307	—	—
Diabetes duration	0.953 (0.917–0.990)	0.014	0.936 (0.895–0.980)	0.005
Mean arterial blood pressure	1.018 (0.993–1.045)	0.164	—	—
HbA1c	0.961 (0.788–1.172)	0.693	—	—
Systemic hypertension	1.058 (0.554–2.019)	0.865	—	—
Dyslipidaemia	0.996 (0.538–1.846)	0.991	—	—
Phakic in both eyes	1.061 (0.549–2.050)	0.861	—	—
PDR in either eye	1.597 (0.747–3.414)	0.227	—	—
Prior PRP in either eye	3.378 (1.583–7.208)	0.002	7.224 (2.712–19.240)	<0.001
Titer of anti-fumarase IgG	970.3 (15.4–61153.5)	0.001	143.5 (1.2–17185.2)	0.042
Titer of anti-hexokinase 1 IgG	100.1 (6.7–1507.3)	<0.001	41.3 (1.8–941.3)	0.020

Univariate logistic regression or Multivariate logistic analysis using significant parameters (Wald’s chi-square > 2) as independent parameters.

## Discussion

In the current study, we identified anti-hexokinase 1 antibody as a novel autoantibody in sera from a subgroup of DME patients. In the screening of anti-retinal antibodies using Western blot analysis, IgG in most DME sera reacted to several autoantigens. Immunoprecipitation and subsequent peptide mass fingerprinting revealed one of the autoantigens to be hexokinase 1, which is expressed in the outer mitochondrial membrane in many cell types^31^. Hexokinase 1 is highly expressed in the OPL and the outer half of the INL in the human and rodent model retinas. Since these layers are composed of several cell types, e.g., bipolar cells, Müller cells, horizontal cells, and vascular cells, we could not determine its subcellular localization in specific cell types. Immunostaining demonstrated that IgG from DME sera often showed fluorescence signals in the OPL and was partially colocalized with hexokinase 1^32^. Quantitative analyses using ELISA revealed that the titer of anti-hexokinase 1 IgG was high in the subgroup of DME cases but not in PDR cases, suggesting the diagnostic significance of this autoantibody.

Serum biomarkers of diabetic complications would have clinical feasibility in the internal medicine clinic, where blood sampling is routinely performed. We thus employed statistical analysis to validate the significance in the diagnosis or screening of center-involved DME. Multivariate analysis identified a higher titer of anti-hexokinase 1 IgG as a predictor of center-involved DME in diabetic patients, suggesting diagnostic significance, as in the case of anti-fumarase IgG^33^. However, 63 patients (75.9%) with DME had lower titers than the threshold, and the sensitivity was not high, which is consistent with low accuracy in the prediction of DME in the AROC analysis. These results mean that this autoantibody was not suitable for the screening of DME. Since Western blot demonstrated serum autoantibodies against several retinal antigens, further research to identify other autoantibodies may facilitate a composite diagnosis of higher quality.

The screening of anti-retinal antibodies using Western blot analysis revealed an autoantigen with an approximate molecular weight of 102 kDa in 7 (70.0%) of the 10 sera samples from DME patients, although ELISA demonstrated that only 20 (24.9%) of the 83 DME serum samples contained titers of anti-hexokinase 1 antibody above the cutoff value (mean + 2 SD). This discrepancy can be explained by other 100–110-kDa autoantigens in addition to hexokinase 1. Anti-retinal antibodies in the sera are induced in several diseases, e.g., cancer-associated retinopathy, melanoma-associated retinopathy, nonparaneoplastic autoimmune retinopathy (AIR), and AMD^19,34–36^. Because specific autoantigens with 100–110-kDa molecular weights have not been reported in these diseases, further investigation may reveal unknown autoantigens. Another possible explanation for the abovementioned discrepancy is that different epitopes were analyzed between sodium dodecyl sulfate-polyacrylamide gel electrophoresis (SDS-PAGE) and ELISA. ELISA maintains the three-dimensional conformation and therefore does not allow autoantibodies to recognize certain cryptic epitopes. By contrast, proteins with one-dimensional amino acid sequences are separated under reducing and denaturing conditions in SDS-PAGE, which means that autoantibodies can react to cryptic epitopes, generally resulting in an immunoblot analysis with higher sensitivity.

It remains to be elucidated whether disrupted retinas induce autoantibodies or whether anti-retinal antibodies promote retinal destruction. Although we have investigated the relationship of anti-hexokinase 1 antibody to systemic and ocular characteristics, we did not determine the mechanisms underlying the induction of this autoantibody. OCT investigations have demonstrated that a few patterns of pathomorphology and cystoid spaces often appear in the INL and OPL^37^. Because hexokinase 1 is highly expressed in the OPL, pathological cellular disruption in the OPL might promote the exposure of this intracellular protein into the extracellular spaces with subsequent antigen recognition. In particular, BRB breakdown may allow retinal antigens to enter into the systemic circulation or let antigen-presenting cells, e.g., monocytes/macrophages, enter and exit the retinal parenchyma^23^. Subsequently, systemic B cells or plasma cells could produce antibodies against hexokinase 1.

Patients with DME but not PDR had higher titers of anti-hexokinase 1 antibody, whereas the titers were not as high in patients with PDR but not DME. In eyes with PDR, the main retinal lesions are the nonperfused areas, in which structural damage is observed in the NFL and ganglion cell layer^38^. We may speculate that this autoantibody is not elicited in PDR sera, because the protein amounts of hexokinase 1 are not high in these layers. Another possibility is that retinal antigens are lost in neurodegenerative processes within the nonperfused areas before the recognition of retinal autoantigens.

We further considered the molecular mechanisms underlying the loss of immune tolerance. The mRNA levels and location of hexokinase 1 expression did not differ between retinas from control and Ins2Akita C57BL/6 J mice, which suggests that changes in the levels or localization of gene expression did not influence the induction of this autoantibody. Instead, we hypothesize that posttranslational modifications, e.g., phosphorylation, glycation, and oxidation, can provide new epitopes and contribute to the loss of immune tolerance because high glucose often induces such modifications^39–41^. In addition, human leucocyte antigens (HLAs) are key molecules used for epitope presentation in antigen-presenting cells, e.g., dendritic cells and microglia/macrophages, and the heterogeneity of HLAs might explain why the titer is increased in the sera from a subgroup of but not in all DME patients. Another possibility is that diabetic complications other than DR may promote cellular damage and concomitant exposure to intracellular autoantigens and could be significantly related to DME.

We further considered the pathogenicity of anti-hexokinase 1 antibody. Because hexokinase 1 is highly expressed in the neuroglial components in the OPL, it is necessary to consider their neurotoxic effects^32^. With vascular hyperpermeability, smaller molecules enter the cytoplasm, which leads to edematous changes, including intracytoplasmic swelling and liquefaction necrosis in Müller cells^42^. Neurotoxic factors in the plasma, e.g., thrombin and plasmin, also disrupt neuroglial components^43^. We speculated that such changes promote the extracellular exposure of intracellular hexokinase 1 and that anti-hexokinase 1 antibody might react to its antigen and exacerbate neuroinflammation. Neutralizing antibodies generally block the function of their antigens, although it remains largely unknown whether this autoantibody in DME sera can inhibit the function of hexokinase 1. HK1^dea^ mice, in which the HK1 gene is ablated, suffer from deformation of the erythrocytes and anemia^44^. Further investigation should be pursued to determine whether anti-hexokinase 1 antibody is related to the rouleaux formation of red blood cells in DR or diabetic anemia.

There was a mild correlation between age and anti-hexokinase 1 antibody in this study. Anti-retinal antibodies were increased in sera from AMD patients, and immunological changes associated with age remain to be investigated^18,19^. Although anti-hexokinase 1 antibody has not previously been reported as an autoantibody in AMD sera, cystoid spaces often develop in the OPL in AMD, and it remains to be investigated whether this autoantibody is increased in AMD sera. In sera from patients with paraneoplastic or nonparaneoplastic AIR, most autoantibodies react to retinal antigens in photoreceptor cells^45^. Because immunosuppressive treatment is effective for AIR, further clinical trials should be planned to elucidate whether immunosuppressive therapies, e.g., steroids, are effective for humoral immunity in DME.

This study had several limitations. The delayed fixation in the human samples cannot guarantee the correct localization and amount of mRNA levels. We therefore used the common mouse model of diabetes which was processed immediately after sacrifice to confirm the expression. These samples showed almost the same localization of hexokinase 1, although we could not completely reveal the gene expression in living retinas of diabetic patients. It had a small sample size and was retrospectively performed using sera from Japanese patients in a single center. In the future, a prospective study with a larger cohort should be planned to validate the generalizability of the findings. Further experiments for epitope mapping should be planned, and the *in vivo* pathogenicity of this autoantibody should also be analyzed.

We identified anti-hexokinase 1 antibody in the sera from a subgroup of DME patients. Our data suggest that this autoantibody may serve as a novel serum biomarker of DME and for the diagnosis of DME in the internal medicine clinic.

## Methods

### Participants and biosampling

Participants were consecutively enrolled at the Department of Ophthalmology of Kyoto University Hospital from July 2014 to December 2015. We collected sera from nondiabetic subjects (no DM group) and patients with type 2 DM who did not receive any treatment for macular lesions and divided diabetic patients into the following three groups: (1) the DM group, patients with type 2 diabetes but not DR; (2) the DR group, patients with DR but not center-involved DME; and (3) the DME group, patients with center-involved DME. The eligibility criteria included the following: (1) patients with type 2 DM and (2) the absence of treatment for macular lesions. The exclusion criteria included the following: (1) other chorioretinal diseases; (2) media opacity affecting fundus examination or OCT imaging; (3) history of intraocular surgery other than cataract surgery; (4) history of cataract surgery or panretinal photocoagulation (PRP) within one year; (5) history of anti-VEGF treatment; (6) any infection at sample collection; or (7) any history of autoimmune disease or malignancy. In the DR group, eyes that met the inclusion but not the exclusion criteria were evaluated according to the international clinical DR disease severity scale^46^. In the DME group, eyes with center-involved DME were evaluated^47^. If both eyes met the inclusion/exclusion criteria, we selected the eye with greater CSF thickness for analysis. Sera were processed and aliquoted within 1 hour after sampling and immediately stored at −80 °C. We avoided any freeze-thaw cycle before sample use.

This study was conducted in accordance with the Declaration of Helsinki after receiving approval from the Kyoto University Graduate School and Faculty of Medicine, Ethics Committee (C845). The study was registered at the UMIN Japan Clinical Trial Registry (UMIN000014015). We obtained written informed consent after providing a full explanation of the nature of this study.

### OCT

After comprehensive examination, best-corrected decimal VA was measured and converted to the logMAR VA. DME was objectively diagnosed by OCT-measured retinal thickness, as described previously^47,48^. Briefly, retinal sectional images in the macula were obtained using the raster scan mode of spectral-domain OCT (Spectralis OCT; Heidelberg Engineering, Heidelberg, Germany). We quantified the mean retinal thickness in the CSF (within 1 mm) of the Early Treatment Diabetic Retinopathy Study (ETDRS) grid. Center-involved DME was diagnosed in eyes with a CSF thickness greater than 320 μm and 305 μm in male and female patients, respectively^47^.

### Animals

Two- to three-month-old male mice (C57BL/6 J wild-type and Ins2Akita diabetic animals) were purchased from SLC Japan (Hamamatsu, Japan) and housed in accordance with the Institutional Animal Care and Use Committee guidelines as well as the Association Research in Vision and Ophthalmology (ARVO) Statement for Use of Animals in Ophthalmic and Vision Research. All protocols were approved by the Institutional Review Board of the Kyoto University Graduate School of Medicine (MedKyo 14564, MedKyo 15197, MedKyo 16102). Following anesthesia with a lethal dose of ketamine/xylazine, eyes were harvested and used for further experiments, e.g., RNA purification and immunostaining.

### PCR

RNA levels in the retinas were evaluated using PCR. Briefly, mouse retinas were harvested, frozen in liquid nitrogen, and stored at −80 °C. RNA from the homogenized retinas was isolated using ice-cold Sepasol RNA I Super solution (Nacalai Tesque, Japan) and chloroform, followed by ethanol precipitation. The purified mRNA was applied to reverse transcription using SuperScript II (Life Technologies, Gaithersburg, MD) in order to obtain cDNA. Human retinal cDNA was purchased from ScienCell Research Laboratories (Carlsbad, CA). Human and mouse cDNA was amplified using Taq polymerase (Ex Taq; TaKaRa Bio, Otsu, Japan) as follows: 95 °C for 20 seconds, 56 °C for 20 seconds, and 72 °C for 30 seconds, for 35 cycles. Forward and revere primers (5′-GTTGGTGTCGACGGATCTCT-3′ and 5′-CGCATCCTCTTCTTCACCTC-3′, respectively) were used to determine the mRNA expression of human hexokinase 1 (accession number: NM_000188) in the retinas. Similarly, mouse cDNA was applied to PCR using forward and reverse primers (5′-AGTGGAAGCCAGCTTTTTGA-3′ and 5′-TTCAGCAGCTTGACCACATC-3′, respectively) for murine hexokinase 1 (accession number: NM_001146100.1). PCR products were subjected to 1% agarose gel electrophoresis with ethidium bromide staining (Nacalai Tesque).

### Immunostaining

Immunohistochemistry was performed as previously described^49^. After fixation in 4% paraformaldehyde for 1 hour at 4 °C, the eyes were serially dehydrated and frozen in OCT compound (Sakura Finetek, Torrance, CA). Cryosections at a thickness of 10 μm underwent staining with IgG from DME sera or an anti-hexokinase 1 antibody. After blocking in 10% donkey serum, sections were incubated with serum specimens from DME patients or control subjects (1:100 in 10% donkey serum) or rabbit anti-hexokinase 1 antibody (1:100, Cell Signaling Technologies, Beverly, MA) at 4 °C overnight. Goat anti-human IgG secondary antibodies conjugated to Cy3 (1:5000, Jackson ImmunoResearch, Westgrove, PA) or anti-rabbit secondary antibodies conjugated to Alexa Fluor 488 (1:5000, Life Technologies) were incubated for 1 hour, followed by counterstaining with diamidino-2-phenylindole (DAPI).

Paraffin-embedded retinal sections of human adult eyes were obtained from BioChain Institute Inc. (Newark, CA). After deparaffinization, the sections were incubated with anti-hexokinase 1 antibody (1:100), followed by the secondary antibodies conjugated to Alexa Fluor 594 (1:5000, Life Technologies). Retinal images were obtained by fluorescence microscopy (BZ-9000; Keyence, Osaka, Japan).

### ELISA

Recombinant human fumarase (1 μg/ml, RayBiotech Inc., Norcross, GA) or recombinant human hexokinase 1 (2 μg/ml, ATGen, Gyeonggi-do, Korea) in 100 mM bicarbonate-carbonate buffer (pH 9.6, 100 μl) was incubated in a 96-well Nunc Immunoplate Maxisorp plate (Thermo Scientific Inc., Waltham, MA) at 4 °C overnight, as described previously^33^. After blocking (3% bovine serum albumin [BSA] in Tris-buffered saline containing 0.1% Tween-20 [TBS-T]), the wells were incubated in serum (1:1000 diluted in 3% BSA in TBS-T) for 2 hours, followed by horseradish peroxidase (HRP)-labeled anti-human IgG secondary antibodies (1:5000) for 1 hour, to quantify total levels of anti-hexokinase 1 IgG. The signals detected using 3,3′,5,5′-tetramethylbenzidine (TMB; Nacalai Tesque) were measured at an absorption wavelength of 450 nm using an ARVO MX plate reader (PerkinElmer, Norwalk, CT). All experiments were performed in duplicate and in a blinded manner, and the individual plates were calibrated using two different control serum specimens.

### Immunoblot Analysis

SDS-PAGE using the NuPAGE system (Life Technologies) was performed as described previously^6^. Eyeballs from dead pigs were stored on ice soon after slaughter. Isolated porcine retina or recombinant human hexokinase 1 was lysed with Laemmli buffer with protease inhibitors. After centrifugation at 15,000 × *g* for 5 minutes, supernatants were separated on a gradient gel (4–12%) and transferred to nitrocellulose membranes.

After the membranes were blocked with 5% milk in TBS-T, they were incubated in the primary antibody, i.e., serum specimens from DME patients (1:200) or anti-hexokinase 1 antibody (1:1000), followed by HRP-labeled anti-human or anti-rabbit IgG secondary antibodies, respectively (1:5000, Life Technologies). Chemiluminescence signals were detected using an enhanced chemiluminescence (ECL) detection system (GE Healthcare, Piscataway, NJ).

### Immunoprecipitation

Serum IgG was conjugated to protein A-coated beads (rmp Protein A Sepharose Fast Flow; GE Healthcare) using a crosslinker (dimethyl pimelimidate [DMP], Thermo Scientific). Porcine retinas were lysed with immunoprecipitation buffer (50 mM Tris, pH 7.4, 1% Nonidet-P 40, 10 mM EDTA, 150 mM NaCl with protease inhibitors) according to a modification of methods described previously^33,50^. The lysate was incubated with Protein A beads crosslinked to IgG from DME serum for 2 hours at 4 °C. Immunoprecipitated proteins were eluted with 0.1 M glycine (pH 2.0) at 4 °C and subjected to SDS-PAGE as described above.

### Mass spectrometry

Proteins in the polyacrylamide gels underwent silver staining using a Pierce Silver Stain for Mass Spectrometry Kit (Thermo Scientific). The stained bands were excised and in-gel-digested with trypsin using an In-Gel Tryptic Digestion Kit (Thermo Scientific). The tryptic digests were separated using nanoflow liquid chromatography (Nano-LC-Ultra 2D-plus equipped with cHiPLC Nanoflex [Eksigent, Dublin, CA, USA]). The eluate was directly introduced into a mass spectrometer (TripleTOF 5600+ System coupled to a NanoSpray III source and heated interface [AB SCIEX, Framingham, MA]) and ionized in an electrospray ionization-positive mode. Data were acquired using an information-dependent acquisition method. The acquired datasets were analyzed by ProteinPilot software, version 4.5 beta (AB SCIEX), with the NCBI nr database (June 2016).

### Statistical analysis

The results are expressed as the mean ± SD. Student’s t-test or one-way ANOVA with Bonferroni correction was applied for the parametric populations. The Kruskal-Wallis test with Dunnett’s multiple comparison test or the Mann-Whitney U-test was performed to evaluate the significance of differences in the population with nonnormal distributions or unequal variance. Spearman’s rank correlation coefficient was used to show statistical correlations. We employed univariate logistic regression analysis using systemic and ocular factors and found the significant parameters (Wald’s chi-square > 2), which were used as independent variables in the multivariate logistic analysis. The AROC was measured to assess the DR status–discriminating power of the serum titer of anti-hexokinase 1 IgG. The AROC is presented as the mean value and 95% CI. These statistical analyses were conducted using commercial software (PASW Statistics, version 22; SPSS Inc., Chicago, IL), and *P* < 0.05 was considered significant.

## Supplementary information

Supplementary Information
