# Supplementary material for: Anti-Hexokinase 1 Antibody as a Novel Serum Biomarker of a Subgroup of Diabetic Macular Edema
Source: Sci Rep. 2019 Mar 18;9:4806. doi: 10.1038/s41598-019-39777-z (PMC6423027; doi:10.1038/s41598-019-39777-z)
Supplement: Supplementary file 1 — Supplementary Information [file 41598_2019_39777_MOESM1_ESM.pdf]

Supplementary Information

Anti-Hexokinase 1 Antibody as a Novel Serum Biomarker of a Subgroup of Diabetic Macular Edema

Tatsuya Yoshitake, MD,[1] *Tomoaki Murakami, MD, PhD,*[1*] *Shin Yoshitake, MD,*[1]
Kiyoshi Suzuma, MD, PhD,[1] *Yoko Dodo, MD,*[1] *Masahiro Fujimoto, MD,*[1]
Shinji Ito, PhD,[2] *Akitaka Tsujikawa, MD, PhD.*[1]

[1]Department of Ophthalmology and Visual Sciences, Kyoto University Graduate School of Medicine, Kyoto, Japan.
[2]Medical Research Support Center, Graduate School of Medicine, Kyoto University, Kyoto, Japan.

Correspondence and requests for materials should be addressed to Tomoaki Murakami, MD, Department of Ophthalmology and Visual Sciences, Kyoto University Graduate School of Medicine, 54 Shogoin-Kawaracho, Sakyo, Kyoto 606-8507, Japan (phone: 81-75-751-3250; fax: 81-75-752-0933; email: mutomo@kuhp.kyoto-u.ac.jp)
E-mail: mutomo@kuhp.kyoto-u.ac.jp.

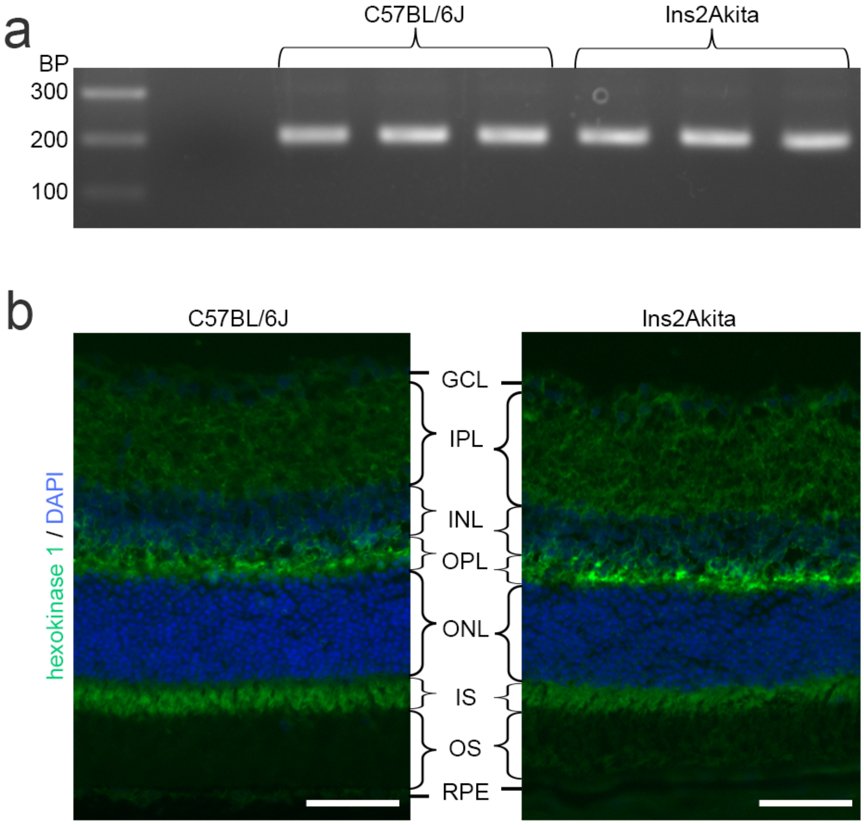

Figure S1: Expression of hexokinase 1 in mouse retinas.

(a) PCR analyses to determine the expression of hexokinase 1 mRNA in retinas from control (C57BL/6J) or diabetic (C57BL/6J Ins2Akita) mice. (b) High levels of hexokinase 1 expression in the OPL and, to a lesser extent, in the IPL in both control and diabetic retinas. Scale bar, 50 µm. GCL, ganglion cell layer; HK1, hexokinase 1; INL, inner nuclear layer; IPL, inner plexiform layer; IS, inner segment; ONL, outer nuclear layer; OPL, outer plexiform layer; OS, outer segment; RPE, retinal pigment epithelium.

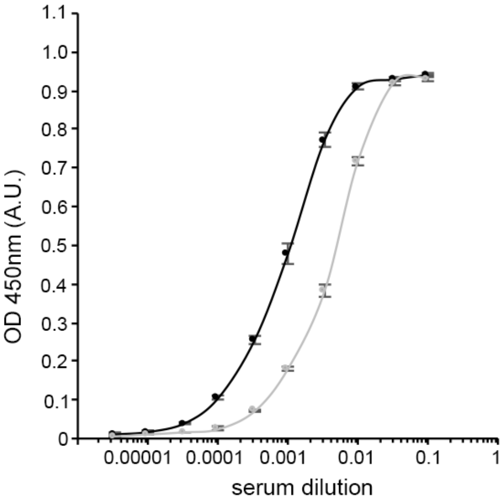

Figure S2: The calibration curves of two representative DME sera in the ELISA.

The patients' characteristics are shown in Supplementary Table S3.

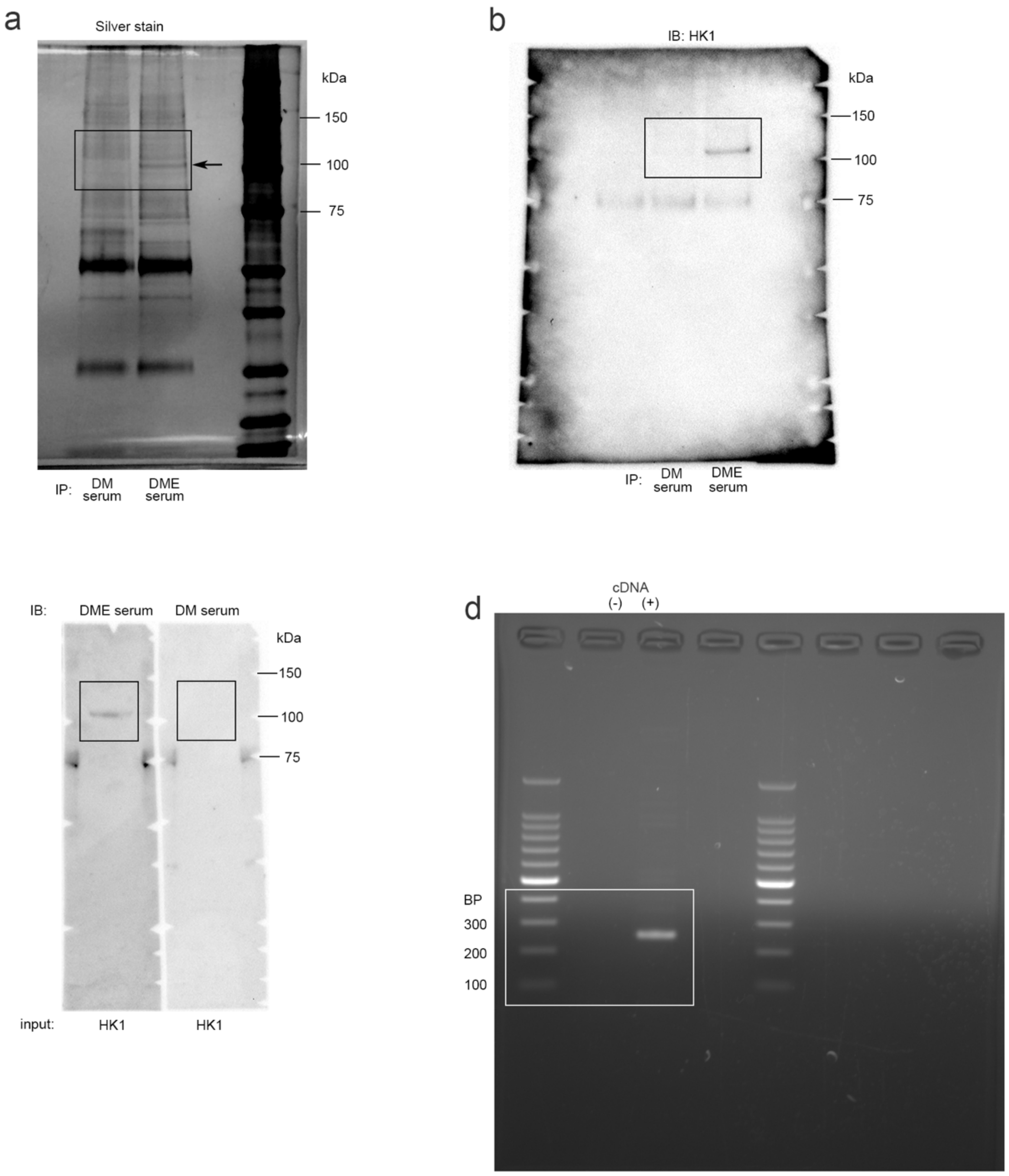

Figure S3. Full unedited gel and Western blot for Figure 2.

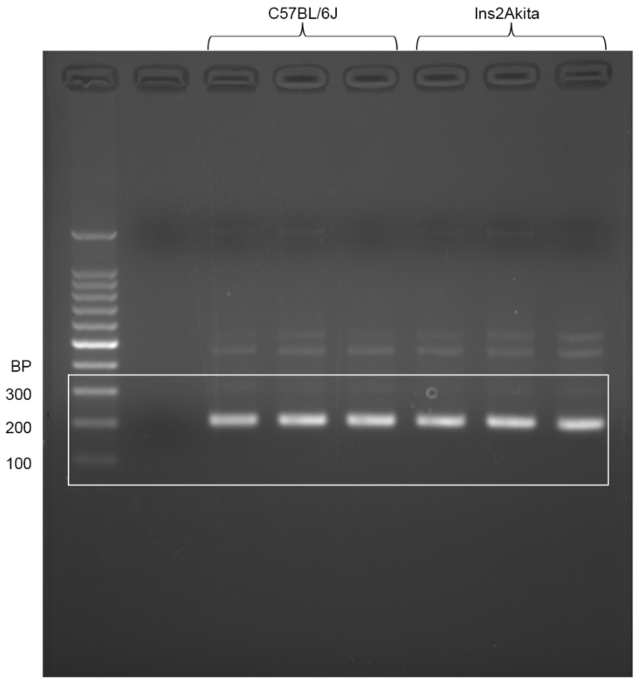

Figure S4. Full unedited gel for Supplementary Figure S1.

Table S1. Characteristics of the patients whose sera were used for immunoprecipitation.

Characteristics	diabetic patient	DME patient
Age (years)	37	72
Gender (male / female)	male	female
Diabetes duration (years)	3	7
Mean arterial blood pressure (mmHg)	96.0	104.3
HbA1c (%)	7.7	7.1
Systemic hypertension	-	+
Dyslipidemia	+	-
Phakic (LE / RE)	+ / +	+ / +
International classification (LE / RE)	-	moderate NPDR / moderate NPDR
Prior PRP in either eye (LE / RE)	-	+ / +

Table S2. Characteristics of the patients whose sera were used for immunofluorescence.

Characteristics	diabetic patient	DME patient
Age (years)	68	72
Gender (male / female)	male	female
Diabetes duration (years)	36	7
Mean arterial blood pressure (mmHg)	88.3	104.3
HbA1c (%)	6.3	7.1
Systemic hypertension	+	+
Dyslipidemia	+	-
Phakic (LE / RE)	+ / +	+ / +
International classification (LE / RE)	-	moderate NPDR / moderate NPDR
Prior PRP in either eye (LE / RE)	-	+ / +

Table S3. Characteristics of two DME patients whose sera were used for the calibration curves.

Characteristics	black line	gray line
Age (years)	62	75
Gender (male / female)	female	male
Diabetes duration (years)	27	25
Mean arterial blood pressure (mmHg)	81.0	107.0
HbA1c (%)	8.5	8.4
Systemic hypertension	-	-
Dyslipidemia	+	-
Phakic (LE / RE)	+ / +	- / -
International classification (LE / RE)	PDR / PDR	severe NPDR / severe NPDR
Prior PRP in either eye (LE / RE)	+ / +	- / -

Table S4. Characteristics of the patients with individual DR severity grades whose sera were quantified using ELISA.

Characteristics	no DR (n=46)	mild NPDR (n=34)	moderate NPDR (n=57)	severe NPDR (n=37)	PDR (n=35)	*P*-value
Age (years)	63.5±11.0	68.2±10.2	64.6±11.5	62.9±12.8	57.3±11.8[††‡]	0.003
Gender (male / female)	32 / 14	22 / 12	38 / 19	28 / 9	24 / 11	0.875
Diabetes duration (years)	11.3±9.8	15.2±9.3	14.2±8.8	13.2±7.8	14.1±10.8	0.394
Mean arterial blood pressure (mmHg)	90.5±7.4	88.5±10.2	96.3±13.7[†]	97.8±11.9[†]	97.8±13.5[†]	0.002
HbA1c (%)	7.76±1.44	7.37±1.21	8.18±1.86	8.10±1.44	7.73±1.61	0.154
Systemic hypertension	25	20	34	24	29	0.092
Dyslipidemia	21	18	32	16	18	0.801
Phakic in both eyes	41	23*	32**	28	28[‡]	0.003
Prior PRP in either eye	-	2	9	10[†]	22[††‡‡§]	<0.001

* *P* <0.05 vs. no DR group; ** *P* <0.01 vs. no DR group; † *P* <0.05 vs. mild NPDR; ††*P* <0.01 vs. mild NPDR group; ‡*P* <0.05 vs. moderate NPDR group; ‡‡*P* <0.01 vs. moderate NPDR group; §*P* <0.05 vs. severe NPDR group.
